# Supplementary figures and images for: Functional NHE1 expression is critical to blood brain barrier integrity and sumatriptan blood to brain uptake
Source: PLoS One. 2020 May 29;15(5):e0227463. doi: 10.1371/journal.pone.0227463 (PMC7259629; doi:10.1371/journal.pone.0227463)

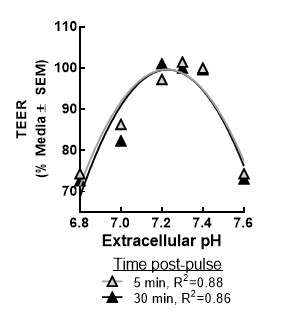

Supplement: S1 Fig — Non-linear regression curve for TEER values at different extracellular pH at 5 and 30 min after the 5 min pH pulse. Values are % of baseline ± SEM (n = 3). (TIF) [file pone.0227463.s001.tif]

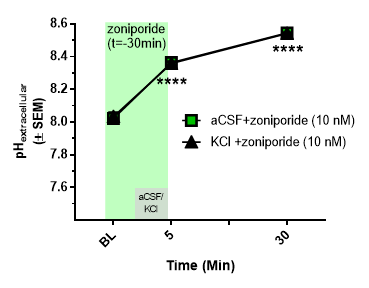

Supplement: S2 Fig — Direct inhibition of NHE1 with zoniporide increased extracellular pH of bEnd.3 cells to prior to aCSF and KCl. No significant difference between aCSF and KCl-treatment at any time-point was observed in these pre-treated cells. Values are mean ± SEM (n = 6). **** p<0.0001 vs. baseline (BL), as assessed by two-way ANOVA with Tukey post-test. (TIF) [file pone.0227463.s002.tif]
