# Supplementary material for: Functional NHE1 expression is critical to blood brain barrier integrity and sumatriptan blood to brain uptake
Source: PLoS One. 2020 May 29;15(5):e0227463. doi: 10.1371/journal.pone.0227463 (PMC7259629; doi:10.1371/journal.pone.0227463)
Supplement: S1 Raw data — (PDF) [file pone.0227463.s003.pdf]

Figure 1A.

TEER	aCSF								
**Baseline**	164	168	165	166	163	161	165	167	168
**post-Pulse**	168	164	165	167	164	166	162	165	165
**10 min**	164	162	165	163	168	169	166	167	168
**20 min**	165	162	163	165	166	167	165	166	165
**30 min**	167	163	166	164	161	163	162	163	169

TEER	aCSF+ zoniporide (10nM)								
**Baseline**	167	163	162	165	166	169	167	162	165
**post-Pulse**	192	194	199	189	194	195	194	189	196
**10 min**	196	191	198	188	196	196	198	197	194
**20 min**	196	191	197	195	192	197	195	193	192
**30 min**	195	196	198	197	198	199	195	189	190

TEER	KCl (60mM)								
**Baseline**	165	168	166	165	164	167	165	164	162
**post-Pulse**	134	132	137	128	131	137	125	132	133
**10 min**	137	135	134	135	136	126	137	132	129
**20 min**	138	134	127	128	137	127	132	134	128
**30 min**	134	136	134	134	130	133	133	133	134

TEER	KCl (60mM)+ zoniporide (10nM)								
**Baseline**	163	166	164	162	164	166	165	162	167
**post-Pulse**	152	148	152	146	153	152	153	152	153
**10 min**	150	152	151	156	154	152	154	157	150
**20 min**	152	153	156	155	154	149	154	151	150
**30 min**	150	146	152	152	149	151	146	147	151

Figure 1B.

	**AUC (mean ± SEM)**
aCSF	660.5 ± 2.957
aCSF+ zoniporide (10nM)	762.8.5 ± 4.242
KCl (60mM)	546.5 ± 5.048
KCl (60mM)+ zoniporide (10nM)	613.6 ± 3.368

Figure 1C.

During pulse: 0-5 min

	% of naïve ± SEM										
**naïve**	90.33	96.08	120.19	93.40	89.94	108.35	101.71	88.48	103.33	103.64	104.56
**aCSF**	105.45	72.73	109.28	98.56	126.00	116.32	75.90	72.24	116.78	98.07	108.89
**KCl**	121.34	125.17	142.97	124.40	189.37	161.29	182.92	140.76	132.87		
**aCSF+zoniporide**	75.22	79.23	80.96	81.91	113.85	111.76	100.38	78.58	83.37	92.34	81.83
**KCl+zoniporide**	106.03	112.15	94.16	100.29	144.02	105.69	157.87	125.91	138.28	131.48	136.74

Figure 1D.

Post pulse: 6-30 min

	% of naïve ± SEM										
**naïve**	93.87	103.38	101.12	101.63	99.22	93.70	107.08	109.33	96.29	101.16	93.21
**aCSF**	105.72	101.28	104.53	98.86	94.64	93.85	95.66	94.63	100.06	105.47	86.30
**KCl**	105.16	90.74	104.17	95.29	97.82	76.18	92.77	100.13	84.07	84.32	76.34
**aCSF+zoniporide**	80.67	77.38	88.56	86.93	90.11	79.63	88.42	84.48	89.38	102.39	101.45
**KCl+zoniporide**	87.13	88.00	70.73	79.45	93.21	90.64	79.89	111.63	98.71	98.18	92.96

Figure 1E.

During pulse: 0-5 min

		% of WT naïve ± SEM								
**wild-type naïve**		108.16	92.58	99.26	110.66	97.44	92.04	116.82	100.76	82.42
**empty vector control**	**media**	203.53	184.79	174.58	88.80	112.15	105.26	128.18	134.24	136.52
	**aCSF**	222.45	212.06	211.32	135.36	119.84	135.63	135.00	121.52	132.58
	**KCl**	226.53	283.30	289.24	185.96	201.48	195.91	215.30	207.58	
***SLC9A1* sgRNA**	**media**	66.42	78.85	77.74	75.84	90.01	83.81	92.73	85.30	83.94
	**aCSF**	89.98	58.63	55.29	92.85	89.74	89.20	87.73	106.97	87.27
	**KCl**	76.07	93.69	95.36	147.91	158.43	139.54	160.61	127.42	126.52

Figure 1F.

Post pulse: 6-30 min

		% of WT naïve ± SEM								
**empty vector control**	**aCSF**	118.06	114.00	129.97	100.17	94.67	102.64	115.30	126.16	124.52
	**KCl**	106.44	120.12	104.47	92.29	149.24	104.10	104.97	115.91	112.41
***SLC9A1* sgRNA**	**aCSF**	104.47	103.62	107.94	79.34	84.17	92.03	97.20	98.28	97.58
	**KCl**	106.64	107.26	92.48	75.16	89.03	91.93	86.59	97.56	87.39

Fig 2B

NHE1

ladder: Precision PlusProtein™ Dual Color Standards, BioRad

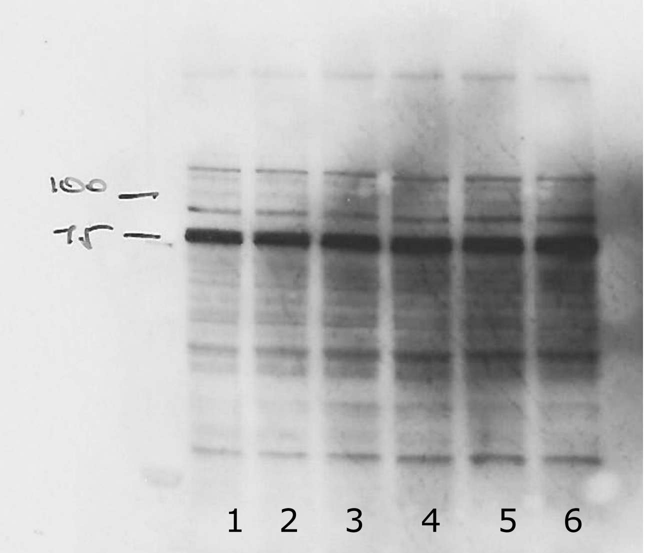

1: Slc9A1 sgRNA
2: Slc9A1 sgRNA
3: empty vector control
4: empty vector control
5: empty vector control
6: empty vector control

alpha tubulin

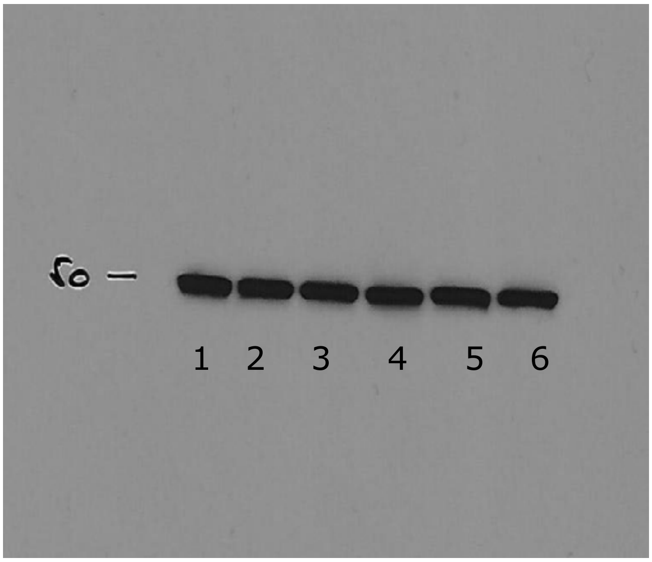

Fig 2B
NHE1
ladder: ThermoScientific, PageRuler Prestained Protein ladder

100 kDa
75 kDa

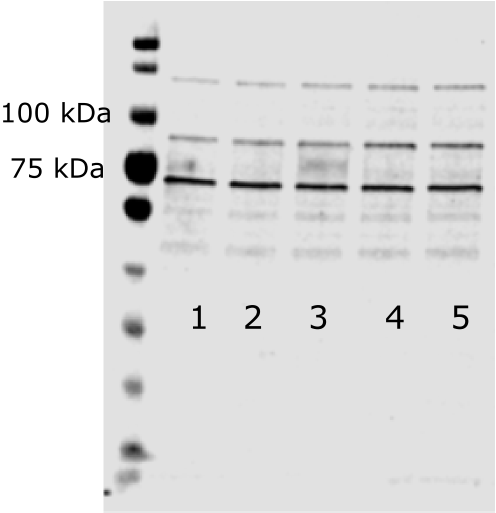

1: Slc9A1 sgRNA
2: Slc9A1 sgRNA
3: Slc9A1 sgRNA
4: empty vector control
5: empty vector control

alpha tubulin

50 kDa

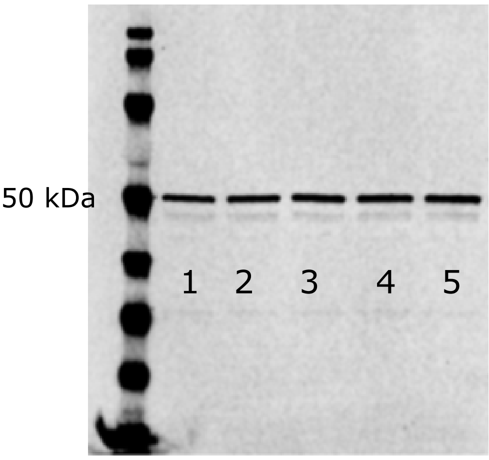

Fig 2B
NHE1
ladder: ThermoScientific, PageRuler Prestained Protein ladder

100 kDa
75 kDa

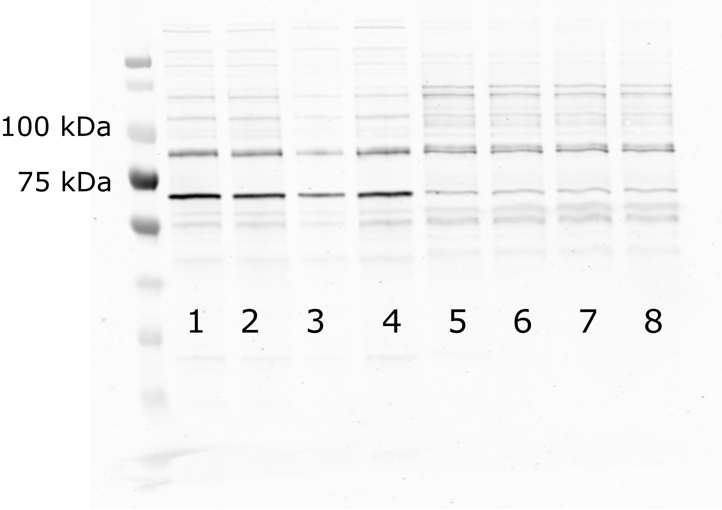

1: empty vector control
2: empty vector control
3: Slc9A1 sgRNA
4: empty vector control
5: Slc9A1 sgRNA
6: Slc9A1 sgRNA
7: Slc9A1 sgRNA
8: Slc9A1 sgRNA

alpha tubulin

50 kDa

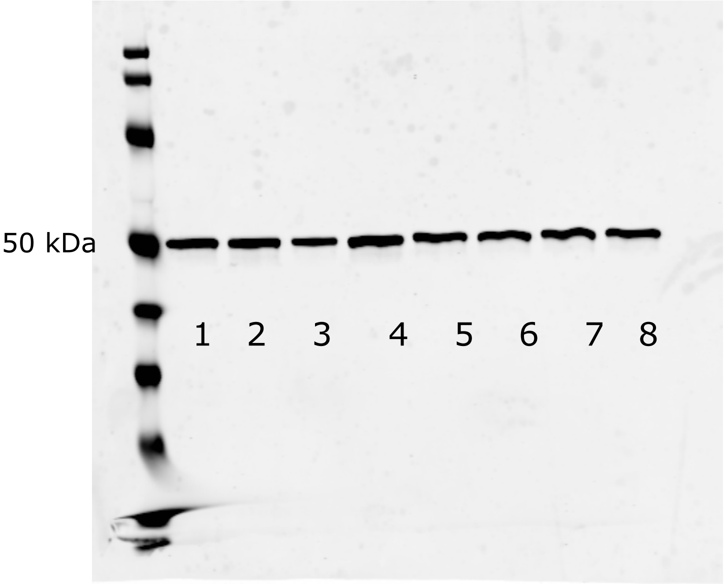

Figure 3A.

During pulse: 0-5 min

	% of naïve ± SEM																	
**naïve**	80.87	114.78	52.17	99.13	120.00	133.04	136.01	74.59	74.59	107.50	95.43	111.88	108.12	91.88	105.06	103.40	95.63	95.90
**aCSF**	122.61	148.70	120.00	120.00	91.31	99.13	109.69	142.60	145.89	182.08	164.53	94.16	83.19	117.09	84.00	83.65	84.41	83.56
**KCl**	146.09	127.83	120.00	143.48	195.65	172.18	152.47	163.44	162.34	172.21	157.95	171.12	115.61	120.37	92.88	130.12	108.88	119.25
**aCSF+zoniporide**	126.81	122.64	93.41	102.94	115.54	104.68	132.68	141.84	130.69	128.15	136.94	137.89	142.66					
**KCl+zoniporide**	151.21	186.43	162.69	165.69	209.74	193.39	182.40	198.50	217.96	204.62	207.58	193.07	150.50					

Figure 3B.

Post pulse: 6-30 min

	% of naïve ± SEM												
**naïve**	140.55	75.62	83.84	82.95	107.36	109.69	100.13	99.87	101.99	94.70	102.30	101.01	
**aCSF**	67.40	131.51	100.27	103.11	78.15	90.36	100.11	97.85	93.50	95.54	101.29	93.35	100.46
**KCl**	101.92	118.60	115.86	98.86	87.17	71.69	92.45	89.53	93.38	89.43	97.34		
**aCSF+zoniporide**	94.67	92.22	91.27	103.30	89.62	82.64	71.69	89.01	95.28	94.59	99.39	95.20	76.68
**KCl+zoniporide**	94.55	91.92	83.71	99.62	88.68	79.88	76.64	85.59	100.78	76.82	91.90	91.90	97.10

Figure 3C.

During pulse: 0-5 min

		% of WT naïve ± SEM												
**wild-type naïve**		84.86	102.52	112.62	97.34	107.87	94.79	111.55	97.98	90.48				
**empty vector control**	**media**	97.40	105.32	106.05	101.21	70.10	122.03	138.57	109.64	111.43				
	**aCSF**	114.20	106.41	125.04	133.41	106.42	145.64	137.14	122.38	134.40				
	**KCl**	145.86	128.33	167.05	207.87	132.81	180.39	179.52	187.62	189.88				
***SLC9A1* sgRNA**	**media**	103.37	105.32	88.76	98.01	81.94	72.32	137.58	132.47	128.33	137.53	149.15	164.40	124.40
	**aCSF**	139.65	136.97	135.88	149.51	147.20	141.84	150.61	104.96	140.80	64.89	171.43	122.74	147.26
	**KCl**	227.80	217.94	190.42	214.53	225.97	200.77	126.87	160.96	220.10	308.11	230.71	241.19	217.50

Figure 3D.

Post pulse: 6-30 min

		% of WT naïve ± SEM												
**empty vector control**	**aCSF**	86.26	95.25	98.53	95.38	100.18	99.58	104.02	86.37	88.86				
	**KCl**	85.54	78.86	94.59	81.38	102.72	87.74	100.26	92.35	97.25				
***SLC9A1* sgRNA**	**aCSF**	131.03	119.46	110.21	125.10	117.62	119.96	111.28	95.70	96.78	101.25	100.51	106.34	92.13
	**KCl**	121.12	120.33	114.38	115.23	108.22	117.85	95.25	95.04	85.82	86.28	97.01	101.44	97.88

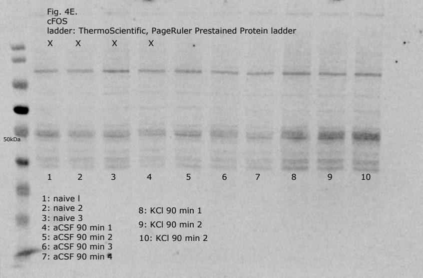
Fig. 4E.
cFOS
ladder: ThermoScientific, PageRuler Prestained Protein ladder
X X X X
50kDa
1 2 3 4 5 6 7 8 9 10
1: naive l
2: naive 2
3: naive 3
4: aCSF 90 min 1
5: aCSF 90 min 2
6: aCSF 90 min 3
7: aCSF 90 min 4
8: KCl 90 min 1
9: KCl 90 min 2
10: KCl 90 min 2

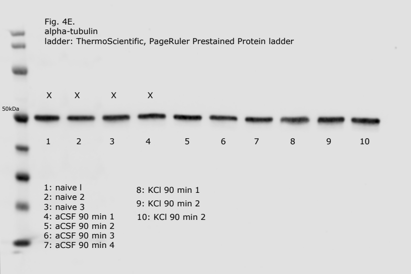
Fig. 4E.
alpha-tubulin
ladder: ThermoScientific, PageRuler Prestained Protein ladder
X X X X
50kDa
1 2 3 4 5 6 7 8 9 10
1: naive l
2: naive 2
3: naive 3
4: aCSF 90 min 1
5: aCSF 90 min 2
6: aCSF 90 min 3
7: aCSF 90 min 4
8: KCl 90 min 1
9: KCl 90 min 2
10: KCl 90 min 2

Figure 5B.

	saline+KCl+sumatriptan					zoniporide+KCl+sumatriptan									
**BL**	8.00	8.00	8.00	8.00	8.00	8.00	8.00	8.00	7.13	8.00	7.39	8.00	8.00	8.00	8.00
**pBL**	1.06	3.32	2.34	1.49	1.00	2.20	2.20	8.00	5.19	8.00	2.10	8.00	2.40	2.40	3.07
**30**	4.09	1.00	1.49	1.00	8.00	2.02	8.00	8.00	8.00	8.00	8.00	8.00	8.00	8.00	8.00
**60**	1.80	1.28	1.00	1.00	8.00	1.00	8.00	8.00	1.49	8.00	1.00	2.77	5.59	8.00	8.00
**90**	1.80	8.00	1.00	1.00	8.00	1.00	8.00	8.00	1.06	8.00	8.00	2.77	8.00	8.00	8.00
**120**	1.00	8.00	1.00	1.00	8.00	1.49	4.95	8.00	8.00	8.00	4.88	2.57	8.00	8.00	1.71
**180**	8.00	8.00	1.00	1.00	8.00	1.49	8.00	8.00	8.00	8.00	8.00	3.41	8.00	8.00	2.15

Figure 5E.

	aCSF					KCl					
**BL**	8.00	8.00	8.00	8.00	6.70	6.74	6.69	6.74	8.00	6.74	6.74
**30**	5.94	8.00	8.00	6.42	2.47	2.28	1.00	1.00	5.61	1.00	3.70
**60**	4.35	8.00	8.00	8.00	2.87	1.00	1.00	1.00	4.79	1.00	2.87
**90**	3.88	3.34	8.00	8.00	2.61	1.00	1.00	1.00	2.87	1.00	1.26
**120**	3.73	1.00	7.86	8.00	3.37	2.90	1.00	1.00	1.87	3.70	2.00
**180**	2.47	1.00	8.00	8.00	3.37	1.00	1.00	2.87	1.00	1.00	1.26

	aCSF+zoniporide						KCl+zoniporide					
**BL**	7.13	8.00	8.00	8.00	8.00	8.00	8.00	8.00	2.00	8.00	8.00	8.00
**30**	3.26	5.58	2.20	2.34	1.06	6.34	8.00	1.00	1.40	7.13	1.89	3.31
**60**	2.44	2.44	1.32	2.05	1.00	2.66	8.00	1.68	1.00	1.28	1.49	2.34
**90**	2.20	1.94	1.00	1.32	1.00	1.80	1.32	1.32	1.00	1.45	1.00	1.00
**120**	5.58	1.32	1.06	1.00	1.66	1.11	1.82	1.70	1.70	1.49	1.43	2.05
**180**	1.32	1.06	1.55	1.06	1.06	1.53	2.62	1.80	1.32	1.00	1.55	1.32

Figure 5F.

	AUC (mean ± SEM)
aCSF	28.52 ± 4.05
KCl	11.96 ± 2.02
aCSF+ zoniporide	13.51 ± 2.04
KCl + zoniporide	13.6 ± 3.13

NHE1, cortex, Figure 6A

ladder: ThermoScientific, PageRuler Prestained Protein ladder

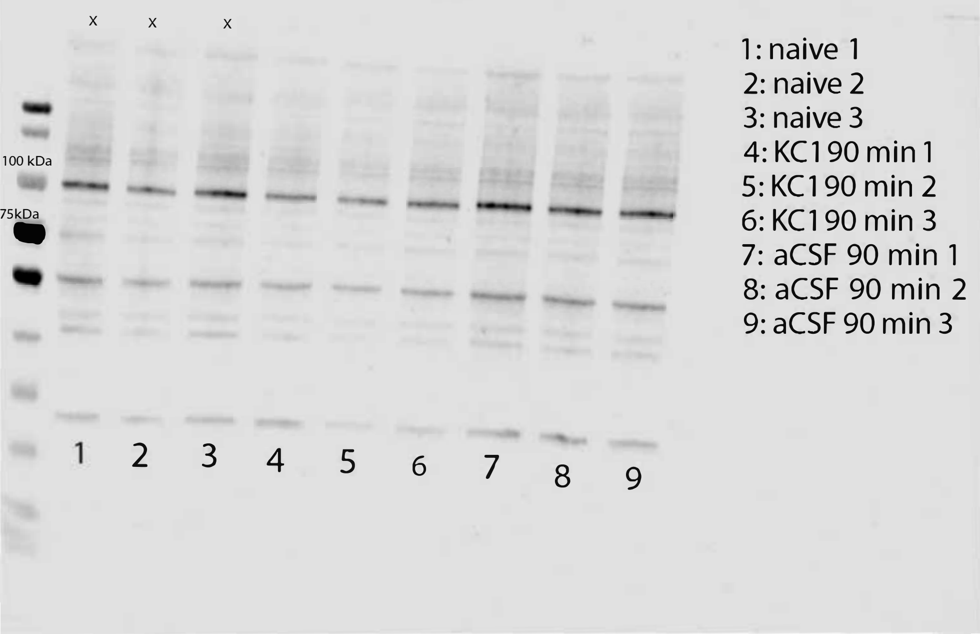

alfa-tubulin, cortex, Figure 6A

ladder: ThermoScientific, PageRuler Prestained Protein ladder

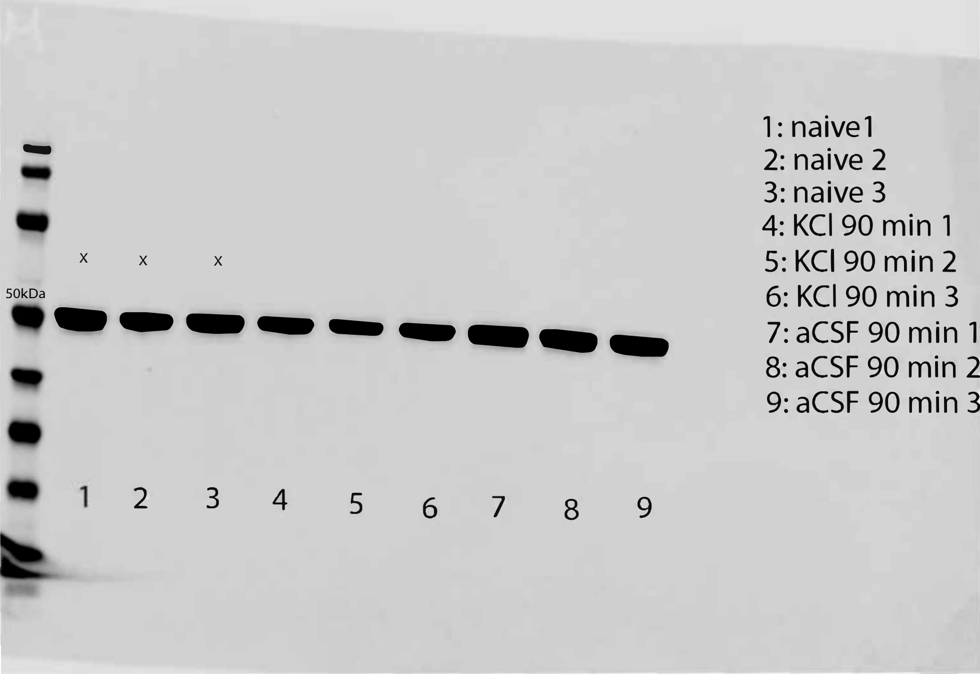

ladder: ThermoScientific, PageRuler Prestained Protein ladder

NHE1, PAG, Figure 6B

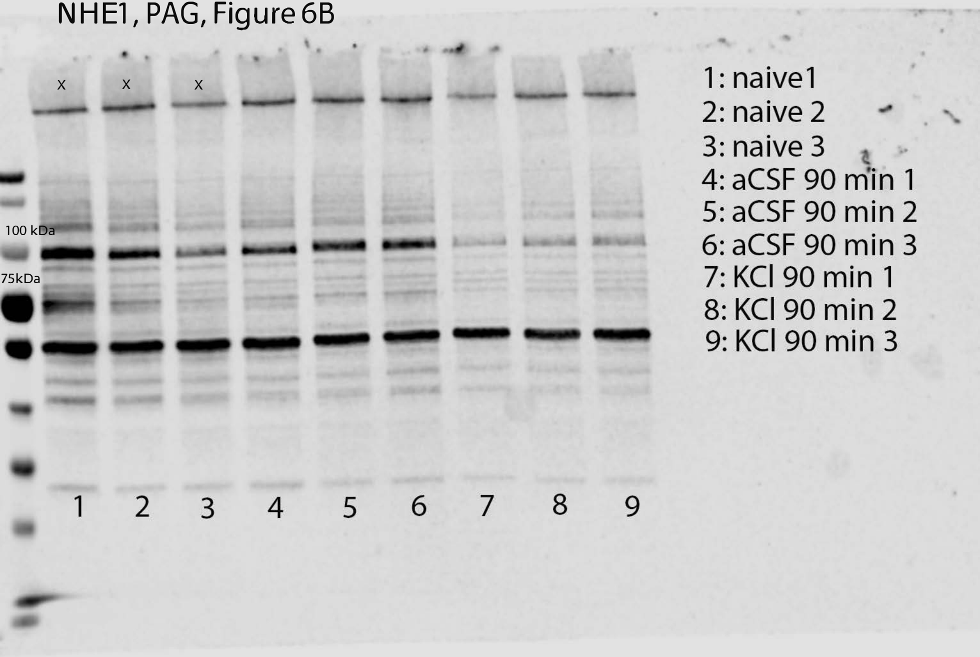

ladder: ThermoScientific, PageRuler Prestained Protein ladder

alfa-tubulin, PAG, Figure 6B

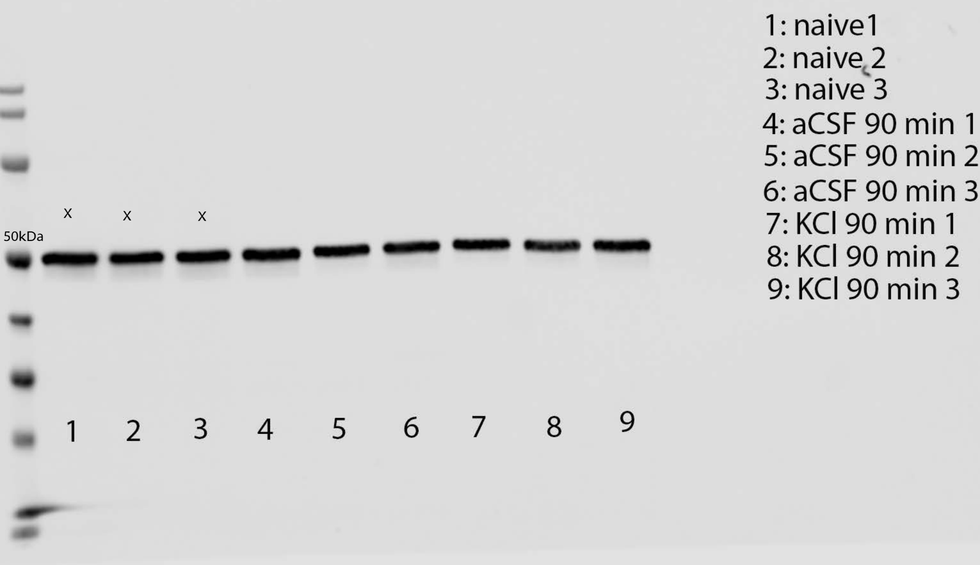

ladder: ThermoScientific, PageRuler Prestained Protein ladder

NHE1, Vc, Figure 6C

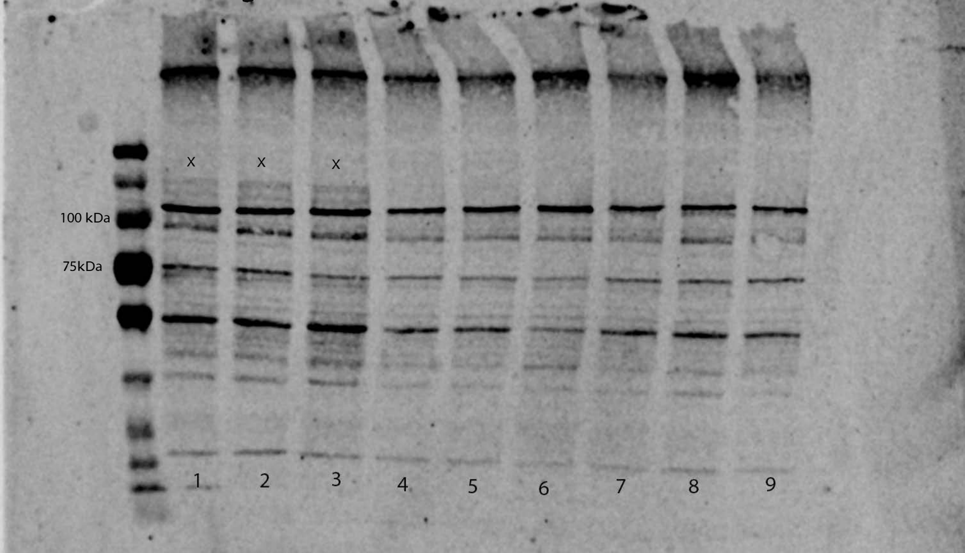

1: naive1
2: naive 2
3: naive 3

4: aCSF 90 min 1
5: aCSF 90 min 2
6: aCSF 90 min 3

7: KCl 90 min 1
8: KCl 90 min 2
9: KCl 90 min 3

[illegible]

ladder: ThermoScientific, PageRuler Prestained Protein ladder

alfa-tubulin, Vc, Figure 6C

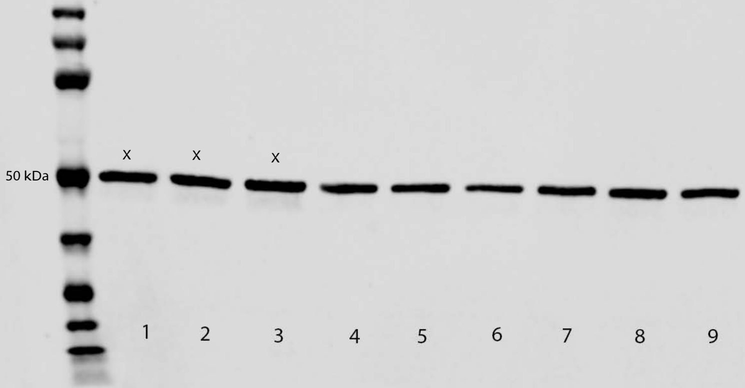

1: naive1
2: naive 2
3: naive 3

4: aCSF 90 min 1
5: aCSF 90 min 2
6: aCSF 90 min 3

7: KCl 90 min 1
8: KCl 90 min 2
9: KCl 90 min 3

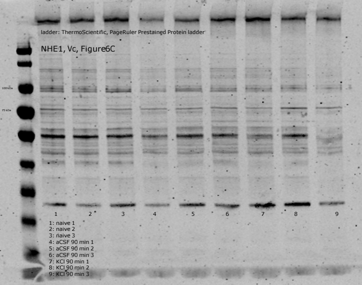

ladder: ThermoScientific, PageRuler Prestained Protein ladder
NHE1, Vc, Figure6C
100 kDa
75 kDa
1
2
3
4
5
6
7
8
9
1: naive 1
2: naive 2
3: naive 3
4: aCSF 90 min 1
5: aCSF 90 min 2
6: aCSF 90 min 3
7: KCl 90 min 1
8: KCl 90 min 2
9: KCl 90 min 3

alpha-tubulin, Vc, Figure 6C.

ladder: ThermoScientific, PageRuler Prestained Protein ladder

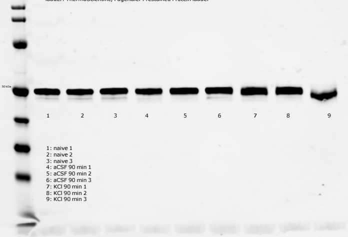

1: naive 1
2: naive 2
3: naive 3
4: aCSF 90 min 1
5: aCSF 90 min 2
6: aCSF 90 min 3
7: KCl 90 min 1
8: KCl 90 min 2
9: KCl 90 min 3

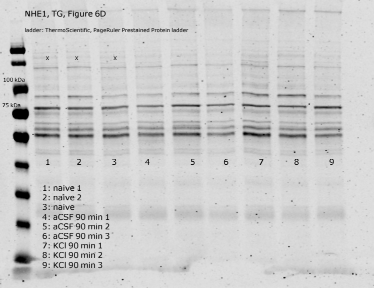
NHE1, TG, Figure 6D
ladder: ThermoScientific, PageRuler Prestained Protein ladder
x
x
x
100 kDa
75 kDa
1
2
3
4
5
6
7
8
9
1: naive 1
2: naive 2
3: naive
4: aCSF 90 min 1
5: aCSF 90 min 2
6: aCSF 90 min 3
7: KCl 90 min 1
8: KCl 90 min 2
9: KCl 90 min 3

alpha-tubulin, TG, Figure 6D

ladder: ThermoScientific, PageRuler Prestained Protein ladder

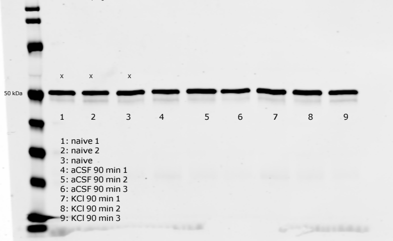

ladder: ThermoScientific, PageRuler Prestained Protein ladder

NHE1, TG, Figure 6D

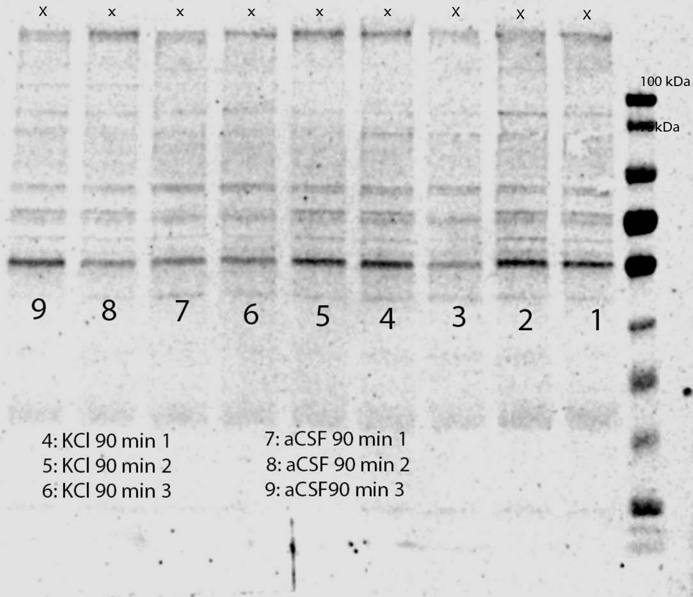

1: naive1
2: naive 2
3: naive 3

4: KCl 90 min 1
5: KCl 90 min 2
6: KCl 90 min 3

7: aCSF 90 min 1
8: aCSF 90 min 2
9: aCSF90 min 3

ladder: ThermoScientific, PageRuler Prestained Protein ladder

alfa-tubulin, TG, Figure 6D

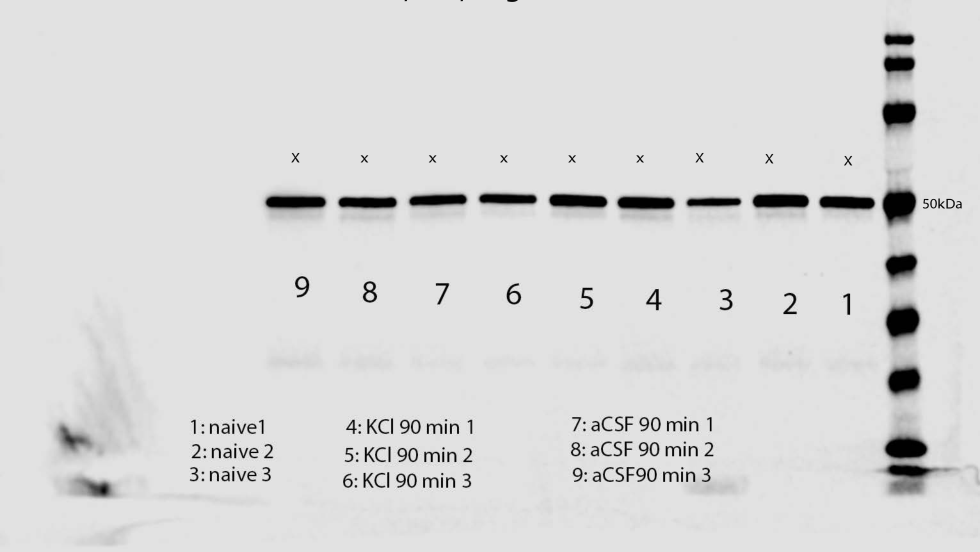

Figure 7C. NHE1 in whole cell lystae

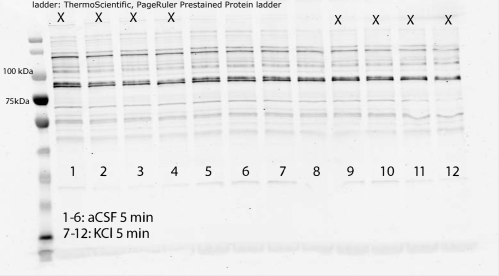

Figure 7C. alpha-tubulin in whole cell lysate

ladder: ThermoScientific, PageRuler Prestained Protein ladder

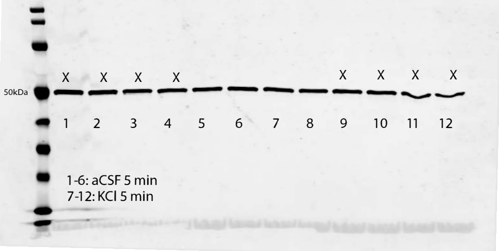

Figure 7B. NHE1

ladder: ThermoScientific, PageRuler Prestained Protein ladder

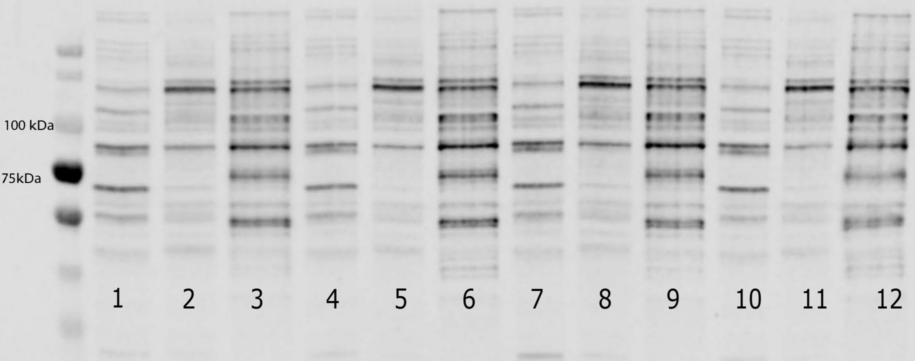

1st set:
1: aCSF 5 min cytosol fraction
2: aCSF 5 min membrane fraction
3: aCSF 5 min nuclear fraction
4: KCl 5 min cytosol fraction
5: KCl 5 min membrane fraction
6: KCl 5 min nuclear fraction

2nd set:
7: aCSF 5 min cytosol fraction
8: aCSF 5 min membrane fraction
9: aCSF 5 min nuclear fraction
10: KCl 5 min cytosol fraction
11: KCl 5 min membrane fraction
12: KCl 5 min nuclear fraction

Figure 7B. alpha-tubulin

ladder: ThermoScientific, PageRuler Prestained Protein ladder

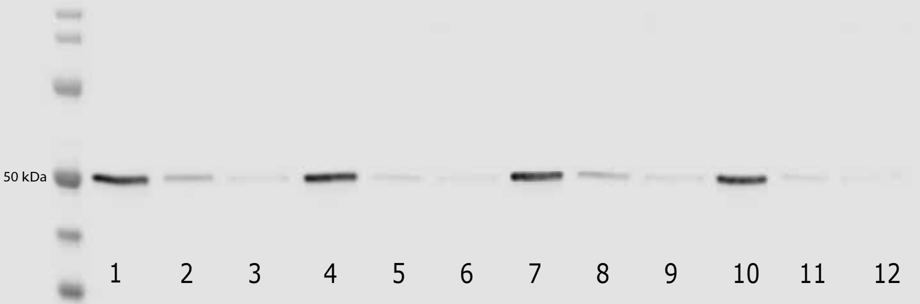

1st set:
1: aCSF 5 min cytosol fraction
2: aCSF 5 min membrane fraction
3: aCSF 5 min nuclear fraction
4: KCl 5 min cytosol fraction
5: KCl 5 min membrane fraction
6: KCl 5 min nuclear fraction

2nd set:
7: aCSF 5 min cytosol fraction
8: aCSF 5 min membrane fraction
9: aCSF 5 min nuclear fraction
10: KCl 5 min cytosol fraction
11: KCl 5 min membrane fraction
12: KCl 5 min nuclear fraction

Figure 7B. lamin B

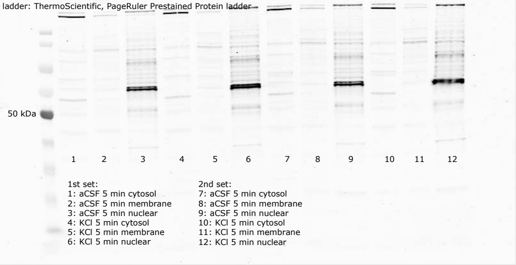

Figure 7B. PECAM

ladder: ThermoScientific, PageRuler Prestained Protein ladder

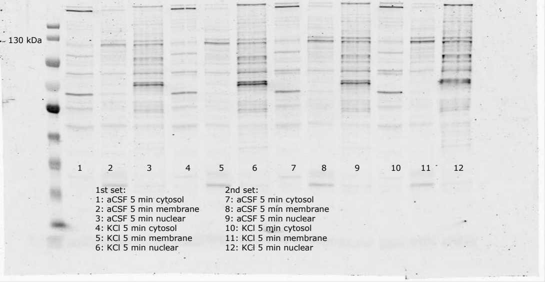

Figure 7B. NHE1

ladder: ThermoScientific, PageRuler Prestained Protein ladder

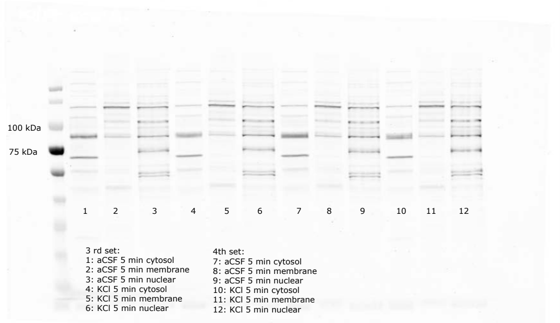

Figure 7B. alpha-tubulin

ladder: ThermoScientific, PageRuler Prestained Protein ladder

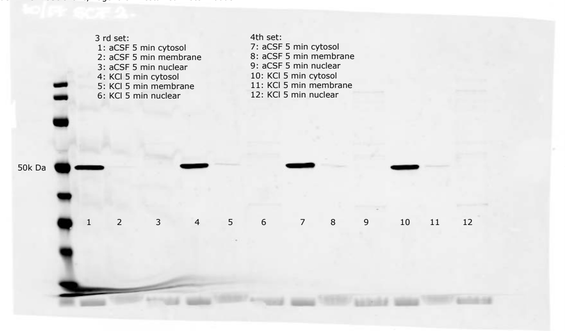

Figure 7B. lamin B

ladder: ThermoScientific, PageRuler Prestained Protein ladder

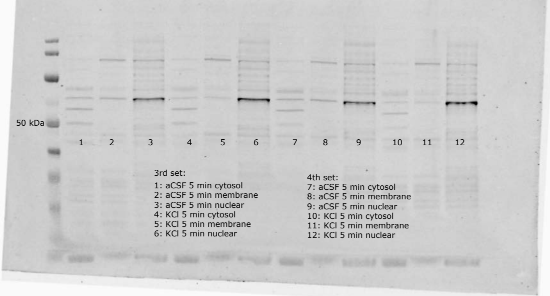

Figure 7B. PECAM

ladder: ThermoScientific, PageRuler Prestained Protein ladder

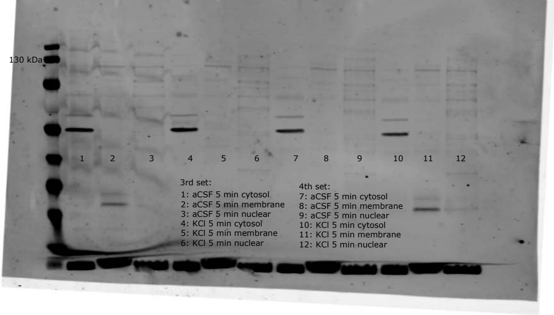

Figure 7B. NHE1

ladder: ThermoScientific, PageRuler Prestained Protein ladder

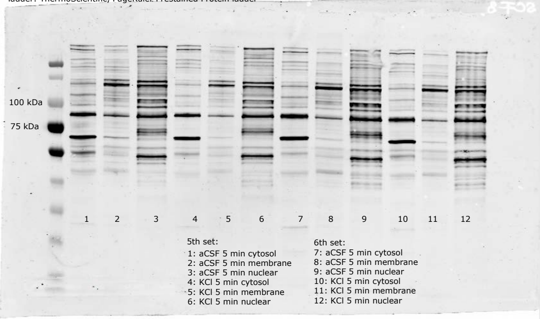

5th set:
1: aCSF 5 min cytosol
2: aCSF 5 min membrane
3: aCSF 5 min nuclear
4: KCl 5 min cytosol
5: KCl 5 min membrane
6: KCl 5 min nuclear

6th set:
7: aCSF 5 min cytosol
8: aCSF 5 min membrane
9: aCSF 5 min nuclear
10: KCl 5 min cytosol
11: KCl 5 min membrane
12: KCl 5 min nuclear

Figure 7B. alpha-tubulin

ladder: ThermoScientific, PageRuler Prestained Protein ladder

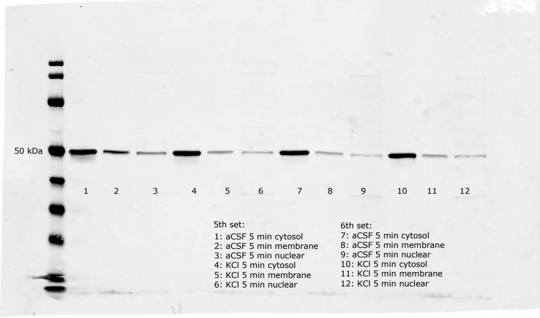

Figure 7B. PECAM and lamin B

ladder: ThermoScientific, PageRuler Prestained Protein ladder

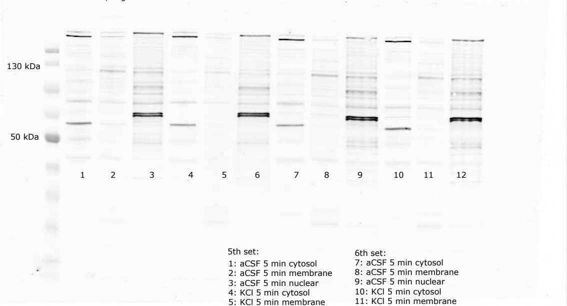

5th set:
1: aCSF 5 min cytosol
2: aCSF 5 min membrane
3: aCSF 5 min nuclear
4: KCl 5 min cytosol
5: KCl 5 min membrane
6: KCl 5 min nuclear

6th set:
7: aCSF 5 min cytosol
8: aCSF 5 min membrane
9: aCSF 5 min nuclear
10: KCl 5 min cytosol
11: KCl 5 min membrane
12: KCl 5 min nuclear

Figure 7B. NHE1
ladder: Precision Plus Protein™ Dual Color Standards, BioRad

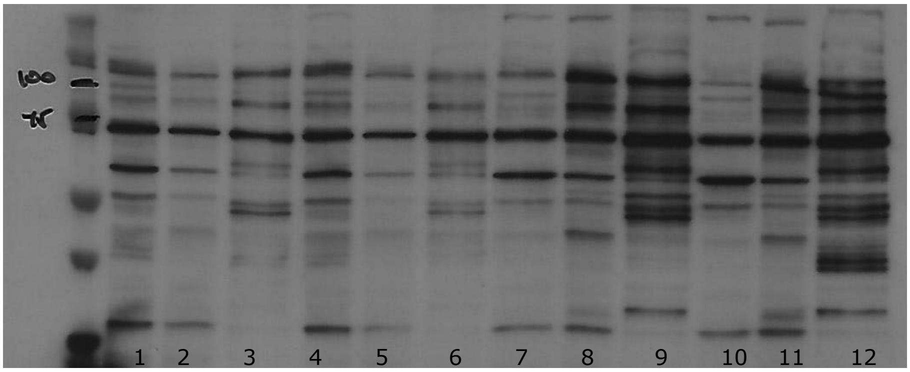

1: aCSF cytosol
2: aCSF membarne
3: aCSF nuclear
4: KCl cytosol
5: KCl membarne
6: KCl nuclear
7: aCSF cytosol
8: aCSF membarne
9: aCSF nuclear
10: KCl cytosol
11: KCl membarne
12: KCl nuclear

alpha-tubulin

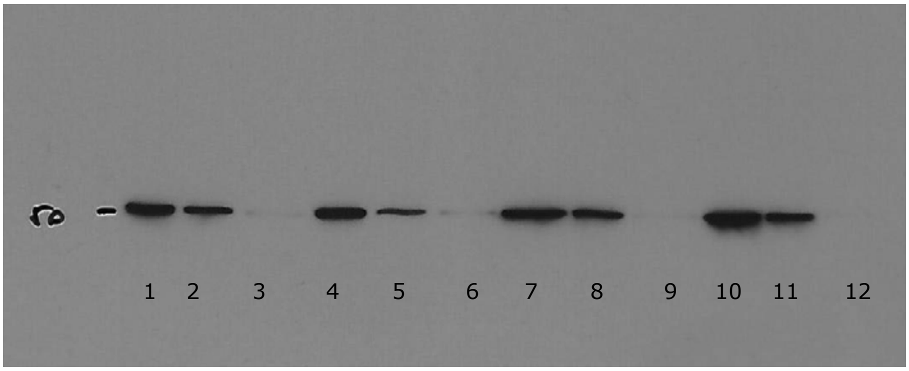

lamin B

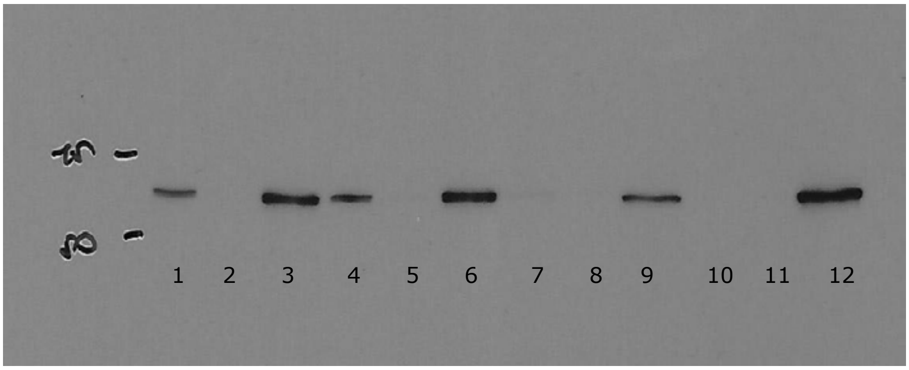

PECAM

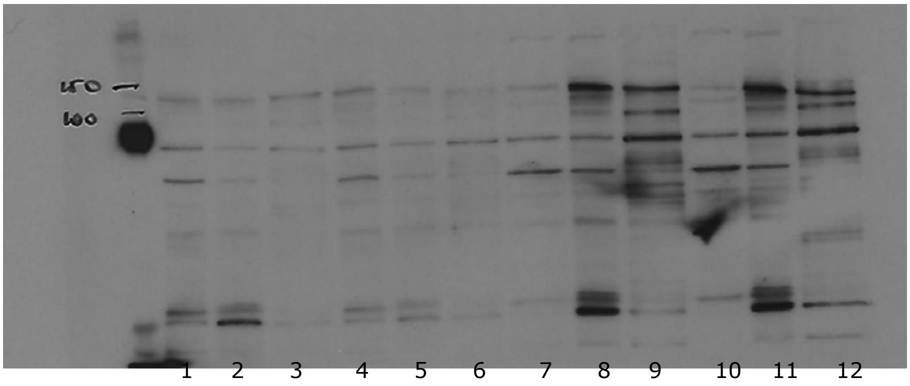

Figure 7B. NHE1
ladder: Precision Plus Protein™ Dual Color Standards, BioRad

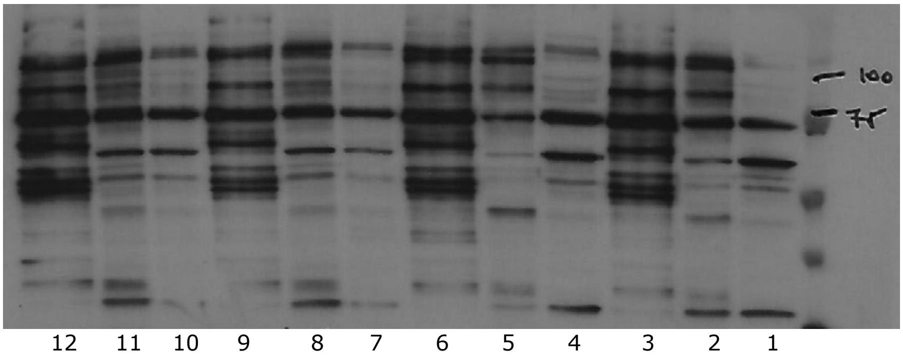

1: aCSF cytosol
2: aCSF membarne
3: aCSF nuclear
4: KCl cytosol
5: KCl membarne
6: KCl nuclear
7: aCSF cytosol
8: aCSF membarne
9: aCSF nuclear
10: KCl cytosol
11: KCl membarne
12: KCl nuclear

alpha-tubulin

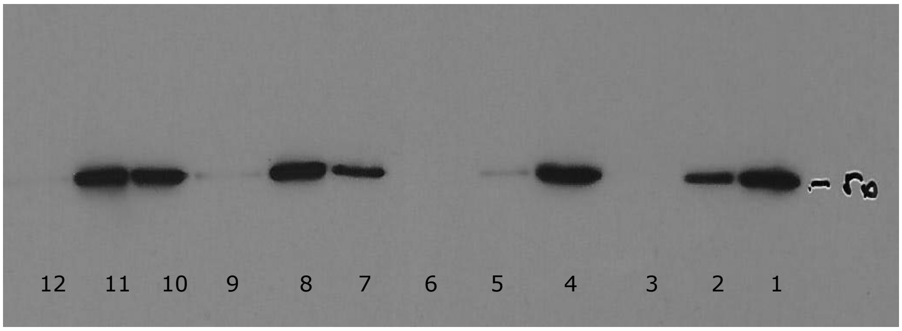

lamin B

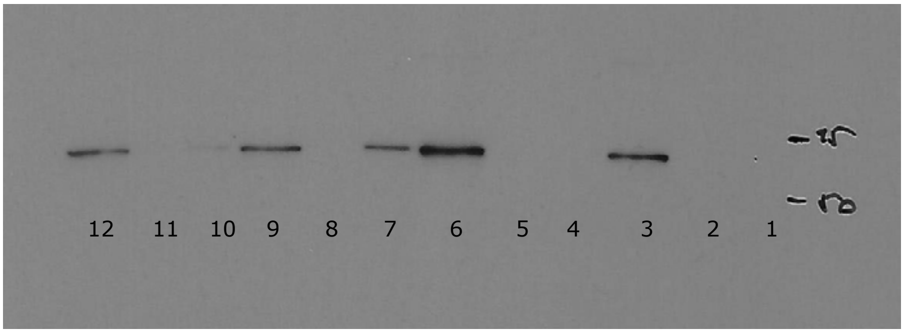

PECAM

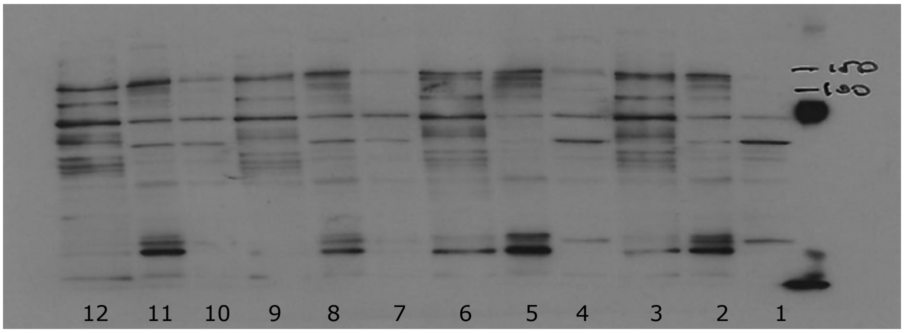

Figure 7B. NHE1
ladder: Precision Plus Protein™ Dual Color Standards, BioRad

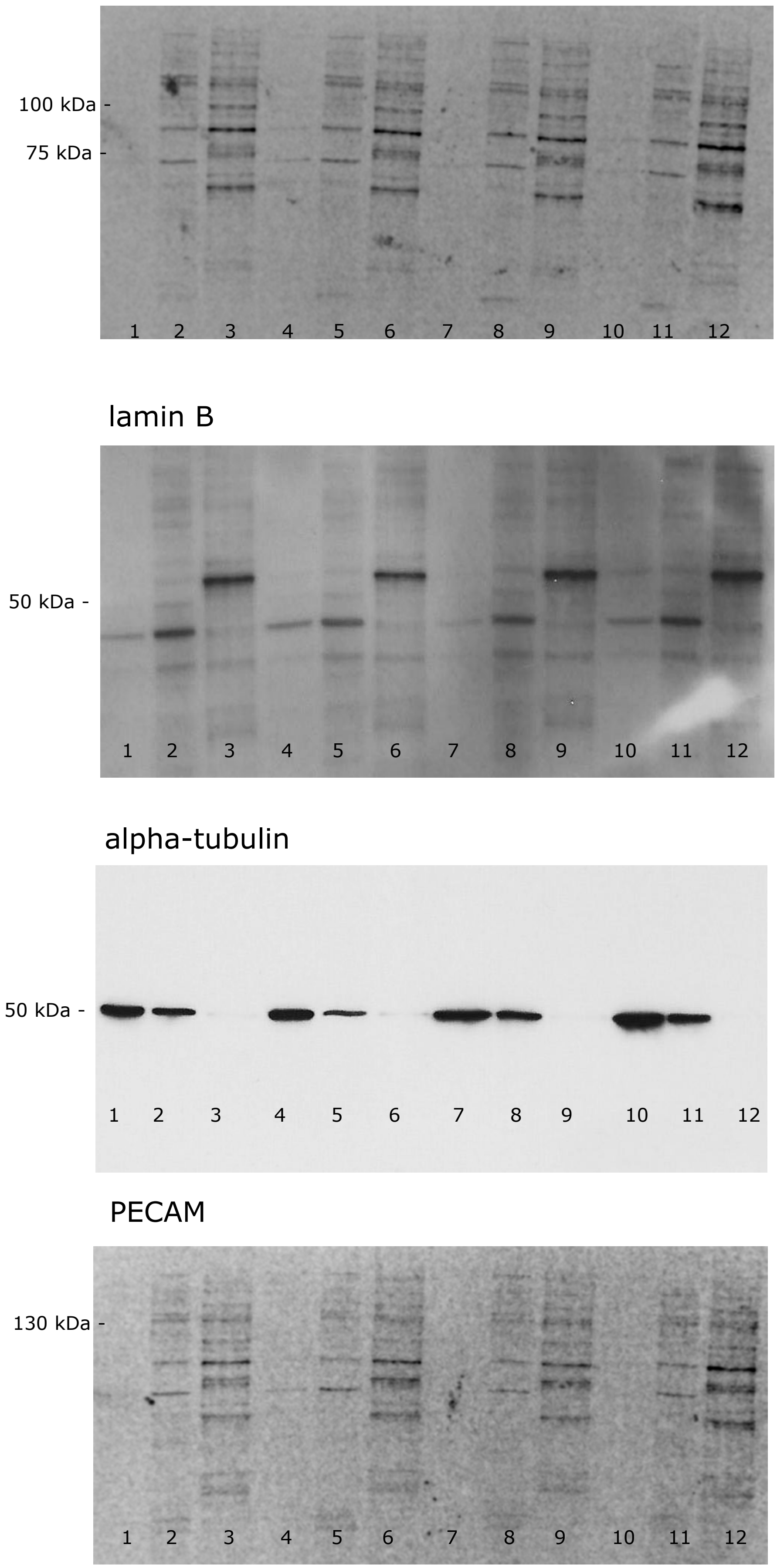

1: aCSF cytosol
2: aCSF membarne
3: aCSF nuclear
4: KCl cytosol
5: KCl membarne
6: KCl nuclear
7: aCSF cytosol
8: aCSF membarne
9: aCSF nuclear
10: KCl cytosol
11: KCl membarne
12: KCl nuclear

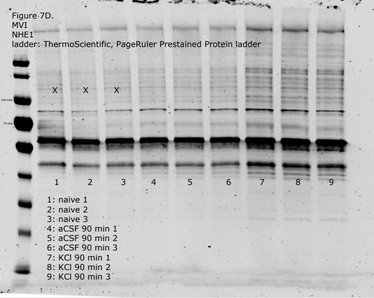
Figure 7D.
MVI
NHE1
ladder: ThermoScientific, PageRuler Prestained Protein ladder
X
X
X
100 kDa
75 kDa
1
2
3
4
5
6
7
8
9
1: naive 1
2: naive 2
3: naive 3
4: aCSF 90 min 1
5: aCSF 90 min 2
6: aCSF 90 min 3
7: KCl 90 min 1
8: KCl 90 min 2
9: KCl 90 min 3

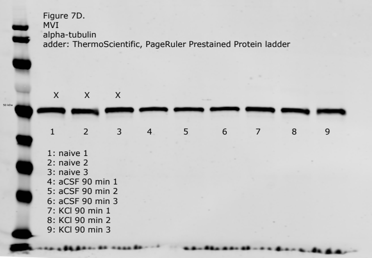
Figure 7D.
MVI
alpha-tubulin
adder: ThermoScientific, PageRuler Prestained Protein ladder
50 kDa
X
X
X
1
2
3
4
5
6
7
8
9
1: naive 1
2: naive 2
3: naive 3
4: aCSF 90 min 1
5: aCSF 90 min 2
6: aCSF 90 min 3
7: KCl 90 min 1
8: KCl 90 min 2
9: KCl 90 min 3

Figure 7E.

	Media		aCSF		KCl (60 mM)	
**Time**	**mean**	**± SEM**	**mean**	**± SEM**	**mean**	**± SEM**
**BL**	6.966	0.147	6.936	0.074	7.273	0.081
**1**	6.821	0.304	7.002	0.077	7.306	0.084
**2**	7.064	0.324	7.025	0.107	6.947	0.109
**3**	6.839	0.260	7.045	0.093	7.525	0.158
**4**	7.232	0.337	7.142	0.089	6.880	0.106
**5**	7.377	0.281	7.315	0.107	6.554	0.156

Figure 7F.

	Media		aCSF		KCl (60 mM)	
**Time**	**mean**	**± SEM**	**mean**	**± SEM**	**mean**	**± SEM**
**BL**	7.682	0.021	7.738	0.019	7.733	0.024
**5**	7.962	0.114	8.140	0.100	8.103	0.103
**30**	7.923	0.090	7.937	0.088	7.952	0.083

Fig 8D

NHE1

ladder: Precision PlusProtein™ Dual Color Standards, BioRad

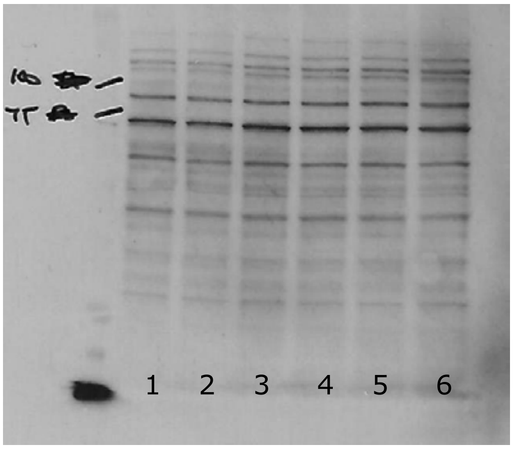

1: naive 1
2: naive 2
3: KCl5 min 1
4: KCl 5 min 2
5: aCSF 5 min 1
6: aCSF 5 min 2

alpha tubulin

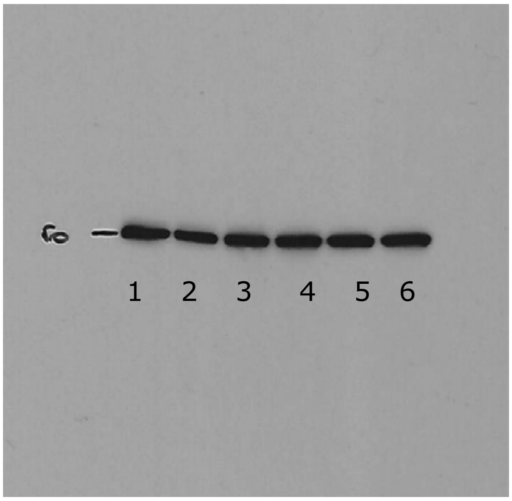

Fig 8E
NHE1
ladder: Precision PlusProtein™ Dual Color Standards, BioRad

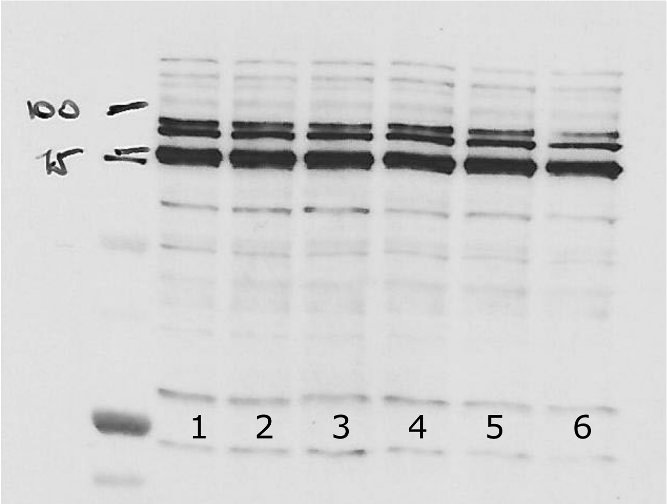

1:naive 1
2: naive 2
3: KCl 5 min 1
4: KCl 5 min 2
5: aCSF 5 min 1
6: aCSF 5 min 2

alpha tubulin

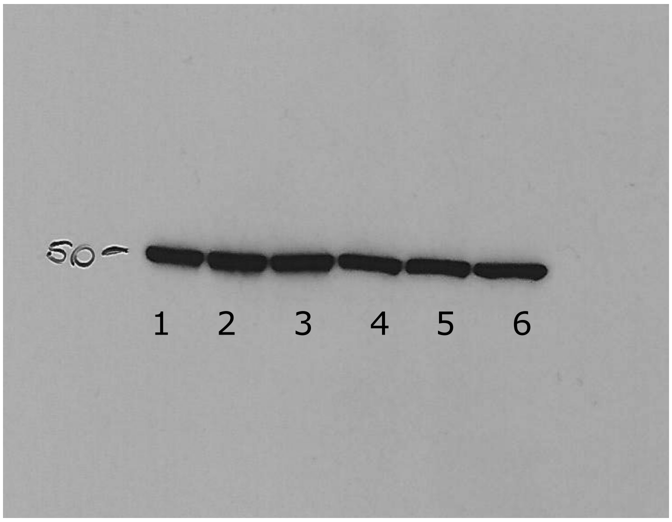

S1 Figure

TEER	Media			6.8			7.0		
**Baseline**	163	161	159	165	163	160	163	160	163
**0**	163	159	160	115	119	119	139	134	137
**10**	163	160	159	111	113	114	133	129	129
**20**	163	163	167	113	115	115	129	131	131
**30**	159	159	157	115	113	117	133	127	131
**60**	165	164	163	113	113	115	131	129	129
**120**	159	161	163	117	114	115	130	129	125
**180**	161	159	160	113	111	115	133	129	129
**360**	163	160	163	111	113	114	129	131	127

TEER	7.2			7.4			7.6		
**Baseline**	163	159	160	159	163	159	161	164	161
**0**	155	153	154	159	159	157	115	119	119
**10**	153	149	155	155	155	159	129	123	127
**20**	155	154	153	159	163	161	115	119	117
**30**	160	163	157	161	155	157	115	117	115
**60**	159	161	159	160	159	159	113	115	114
**120**	163	160	161	160	159	159	115	119	117
**180**	163	160	159	157	155	157	115	117	115
**360**	159	159	163	163	157	155	115	113	114

S2 Figure

pHe	aCSF+zoniporide (10 nM)						KCl +zoniporide (10 nM)					
baseline	7.95	7.96	8.02	8.05	8.08	8.07	7.95	7.96	8.07	8.08	8.07	8.07
5 min	8.31	8.34	8.37	8.38	8.38	8.4	8.32	8.33	8.36	8.37	8.38	8.39
30 min	8.54	8.55	8.55	8.56	8.53	8.54	8.54	8.56	8.54	8.55	8.53	8.53
