# Supplementary material for: Functional NHE1 expression is critical to blood brain barrier integrity and sumatriptan blood to brain uptake
Source: PLoS One. 2020 May 29;15(5):e0227463. doi: 10.1371/journal.pone.0227463 (PMC7259629; doi:10.1371/journal.pone.0227463)
Supplement: Attachment — Submitted filename: Response to Reviewers.docx [file pone.0227463.s004.docx]

Re: Revision of PONE-D-19-34794

Title: Functional NHE1 Expression is Critical to Blood Brain Barrier Integrity and Sumatriptan Blood to Brain Uptake

Response to Referees: The authors wish to thank the reviewers for their remarks and support for the research/manuscript. Below we have addressed individual concerns/comments in **bold**. *Italicized* points are authors’ interpretation of key reviewer concerns. Within the text of the manuscript, we have highlighted revision in red font.

**Reviewer #1:**

**A)** The following statement outlining the overall goal of the study “Given that pH is a known regulator of 1) BBB integrity [30-32] and 2) transport of xenobiotics across the BBB [33,34] and that CSD induces regional perturbations in pH [35], the overall goals of the studies herein focused on NHE1 as a molecular target implicated in cortical KCl-induced deficiencies in BBB integrity resulting from pH dysfunction and regulation of sumatriptan blood to brain uptake” is challenging to parse out. It would be helpful to clarify in series the specific hypothesis of the paper.

**Answer: As the reviewer suggested, we clarified the specific hypothesis in the revised version of the paper.**

**B)** As I understood it, the work is primarily focused on the role of NHE1 on the BBB integrity. However, the bulk of the mechanistic observations are made using cell culture assays, not in freshly dissected intact tissue or in vivo. There is a certain value of these model systems. Induction of depolarization with KCl pulse along with a sucrose assay in endothelial cell monolayers is robust and reproducible. Having said that, this simplistic model system fails to replicate the precise architecture of the endothelial/pericyte system of the intact BBB and how it may be affected in the pathology. ***1****) This limitation is my major concern and at least should be acknowledged and addressed in the Discussion*. At best, the authors may want to address this *by* ***2****) adding new experiment within a section starting on line 420 by testing the BBB integrity in vivo with either live Evan Blue assay or postmortem evaluation of plasma albumin, following the induction of CSD with KCl*. I understand that these are extensive and technically challenging experiments that may be outside the scope of this work. Nevertheless, these would establish a direct link between CSD –BBB failure – a therapeutic action of NHE1 blockade.

**Answers:**

**B1) We agree with the reviewer that in vitro model systems of BBB are not able to fully replicate the precise architecture of the intact BBB. Despite their limitations, several different forms of cell-based systems are widely used and accepted model of BBB. In the current study, we used endothelial cells cultured in astrocyte-conditioned media in order to promote endothelial growth, differentiation and mimic the in vivo neurovascular unit. Our former data (Cottier et al. Loss of Blood-Brain Barrier Integrity in a KCl-Induced Model of Episodic Headache Enhances CNS Drug Delivery. eNeuro 2018; 5:ENEURO.0116-18.2018.) indicated that cortical KCl injection induced paracellular BBB leakiness to radiolabeled sucrose in the cortex, as assessed by in situ brain perfusion. We also observed an increase in BBB permeability to sumatriptan in both the cortex as well as the brainstem. The elevated permeability of sucrose and sumatriptan, observed in those experiment corresponded well with our in vitro data, presented in the current manuscript.**

**B2) We appreciate the recommendation of the reviewer for “adding new experiment within a section starting on line 420 by testing the BBB integrity in vivo with either live Evan Blue assay or postmortem evaluation of plasma albumin, following the induction of CSD with KCl”. We have previously found in Cottier et al., citation above, that paracellular permeability to sucrose was detected. The ringer in this experiment contained Evan’s Blue Albumin; parenchymal uptake of EBA was not detected after cortical KCl injection to induce CSD. To delineate the link between CSD-BBB failure and NHE1 blockade as requested, we investigated FITC-dextran (4000 Da) transport in aCSF and KCl injected rats in the presence of either vehicle or zoniporide. Tissue was harvested 90 after cortical injection for consistency with other data in the current submission as well as Cottier et al., 2018. Parenchymal detection of FITC fluorescence in tissue samples was below the limit of detection (20 ng/mL) for all groups (naïve, saline-aCSF, saline-KCl, zoniporide-aCSF, and zoniporide-KCl), which suggest that BBB breaches *in vivo* after cortical KCl injections do not allow passage of molecules larger than 4kDa (please see method at line 149, results at line 535).**

**Moreover we investigated the expression level of NHE1 in cortical microvessels, isolated from naïve, aCSF, and KCl-treated animals (see in Fig 7D). We did not observe significant differences between aCSF and KCl-treated animals, but the total expression of NHE1 was significantly increased 90 min after cortical KCl injection compared to naïve.**

**C)** Not clear the meaning of the lines 87-88: “…highlights that comprise of NHE1 may have differential effects on BBB 88 integrity versus xenobiotic uptake.”

**Answer: We refined this sentence in the revised version.**

**D)** Lines 357. Please provide a premise for using a sucrose uptake assay to test BBB integrity in endothelial cell monolayer.

**Answer: As mentioned above, radiolabeled sucrose was used in our former, in situ brain perfusion study (Cottier et al., 2018) to investigate changes in BBB paracellular permeability after CSD induction. Sucrose was applied in those and the current study as well, as a model molecule without known transcellular transporter. Therefore, it can represent the paracellular transport, and the paracellular leakage of BBB. We stated the rationale of using sucrose in the revised version of the manuscript.**

**Reviewer #2**:

A) “However, I have a minor concern on the expression of NHE1 in Figure 4C and 4D. It would be in a better readable form if the authors replace these western blotting figures.”

**Answer: As the reviewer suggested we replaced the representative Western blots within Fig 6C and 6D (Original Fig. number is 4, new number is 6). We would like to note that after quantification of a new set of TG samples, the expression of NHE1 after KCl treatment was significantly increased, as we indicated in the graph.**

B) “Another concern is that the authors have given 4 supplementary figures. If the journal has an option to give these figures as main figures, it would be better to include as main figures.”

**Answer: Having consulted the editorial office on the policy for Supplemental Information, we have moved the former S2, S3, and S4 figures to main figures and left S1 and S5 as supplemental figures.**

We would like to note that our blot/gel image data with other raw data can be found in Supporting Information file.
